# Predicting outcomes of acute kidney injury in critically ill patients using machine learning

**DOI:** 10.1038/s41598-023-36782-1

**Published:** 2023-06-18

**Authors:** Fateme Nateghi Haredasht, Liesbeth Viaene, Hans Pottel, Wouter De Corte, Celine Vens

**Affiliations:** 1 KU Leuven, Campus KULAK - Department of Public Health and Primary Care, Etienne Sabbelaan 53, 8500 Kortrijk, Belgium; 2 ITEC - imec and KU Leuven, Etienne Sabbelaan 51, 8500 Kortrijk, Belgium; 3 Department of Nephrology, AZ Groeninge Hospital, President Kennedylaan 4, 8500 Kortrijk, Belgium; 4 Department of Anesthesiology and Intensive Care Medicine, AZ Groeninge Hospital, President Kennedylaan 4, 8500 Kortrijk, Belgium

**Keywords:** Machine learning, Prognosis, Computer science

## Abstract

Acute Kidney Injury (AKI) is a sudden episode of kidney failure that is frequently seen in critically ill patients. AKI has been linked to chronic kidney disease (CKD) and mortality. We developed machine learning-based prediction models to predict outcomes following AKI stage 3 events in the intensive care unit. We conducted a prospective observational study that used the medical records of ICU patients diagnosed with AKI stage 3. A random forest algorithm was used to develop two models that can predict patients who will progress to CKD after three and six months of experiencing AKI stage 3. To predict mortality, two survival prediction models have been presented using random survival forests and survival XGBoost. We evaluated established CKD prediction models using AUCROC, and AUPR curves and compared them with the baseline logistic regression models. The mortality prediction models were evaluated with an external test set, and the C-indices were compared to baseline COXPH. We included 101 critically ill patients who experienced AKI stage 3. To increase the training set for the mortality prediction task, an unlabeled dataset has been added. The RF (AUPR: 0.895 and 0.848) and XGBoost (c-index: 0.8248) models have a better performance than the baseline models in predicting CKD and mortality, respectively Machine learning-based models can assist clinicians in making clinical decisions regarding critically ill patients with severe AKI who are likely to develop CKD following discharge. Additionally, we have shown better performance when unlabeled data are incorporated into the survival analysis task.

## Introduction

Acute kidney injury (AKI) is a very common complication in patients in the Intensive Care Unit (ICU), with up to 50% of patients having the condition^1^. A sudden increase in serum creatinine (SCr) and a decrease in urine volume are characteristic signs of this condition^2^. It is well established that AKI is strongly and independently related to short- and long-term outcomes, such as acute kidney disease (AKD), chronic kidney disease (CKD), and mortality^3^. Even though there have been considerable advances in the treatment of acute kidney injury, the outcomes, particularly in the severe stages, remain poor, with mortality levels often exceeding 50% and some survivors remaining dependent on renal replacement therapy (RRT) for a long period^3–5^.

It has been reported that less than one-third of patients with AKI treated with RRT have their kidney function monitored by a nephrologist after surviving an episode of AKI^6^. The earlier CKD is diagnosed after AKI, though, the less intensive utilization of resources and the better the prevention of morbidity and mortality. Rather than following up with all AKI survivors after hospital discharge, it would be useful to identify subgroups of patients who are at higher risk for negative outcomes and follow them only. It is, therefore, necessary to develop prediction models in order to create a risk score at discharge time, that can be used to determine patient outcomes. Li et al.^7^ developed prediction models for mortality in critically ill patients with AKI at 90 days and one year following the initiation of RRT. The study found that routinely collected features at the time of RRT initiation are limited in their ability to predict mortality among critically ill patients with AKI receiving RRT. In a separate study with a similar cohort, Järvisalo et al.^8^ developed and validated new prediction models for ICU and hospital one-year mortality customized for patients with RRT-dependent AKI in which the developed models showed acceptable external validity in a validation cohort.

While some studies have proposed models that predict mortality after AKI, the prediction of other negative outcomes, such as CKD following AKI in critically ill patients, is an important but rather understudied area of research. Our literature study only yielded a study protocol for a multicenter prospective observational study, which predicts the occurrence of CKD at 3 years after patients suffered AKI during their ICU stay^9^. Possibly, the lack of such research is due to the fact that the follow-up of AKI survivors is considerably challenging after discharge from the ICU. The reason for this can be attributed to two primary factors. Firstly, the process is time-consuming and costly, and secondly, there is a high rate of dropouts. This can lead to reduced data collection and, in turn, can complicate subsequent analyses. This scarcity of labeled training data presents a key challenge in training supervised learning models in the biomedical field. There is, however, often plenty of unlabeled data available for patients with similar characteristics and background information, e.g., from patients who fall outside the study period but otherwise fulfill all inclusion and exclusion criteria. In this article, we propose to take advantage of such unlabeled data by using it to expand the training set.

It is noteworthy that conventional statistical models, such as Logistic Regression, were the most used approaches in the literature to predict the outcomes of AKI. Nowadays, in the healthcare domain, machine learning algorithms have become increasingly popular due to their ability to handle large amounts of high-dimensional data, which is common in healthcare settings^10^. Electronic health records (EHRs) are capable of storing a large number of features and types, enabling accurate and reliable prediction models to be developed^11^. Furthermore, these machine learning models are capable of capturing complex interactions between the features in the datasets^12^. While there has been progressing in leveraging EHR data for predictive modelings, such as in our study, it is important to acknowledge that there is still room for further exploration and development of machine learning models that fully capitalize on the diversity and abundance of EHR-derived data.

Several machine learning models are currently being used to predict AKI^13–16^. While a study developed predictive models for AKI stage 3 progression among critically ill patients who experienced AKI stage 1/2 using machine learning techniques^17^, no such machine learning prediction models have been constructed for the prediction of CKD after experiencing AKI in the ICU.

In summary, this study addresses the prediction of two important outcomes in critically ill patients who experienced AKI stage 3 during their ICU stay: the development of chronic kidney disease (CKD) and mortality. By employing machine learning-based prediction models, we aim to provide valuable insights into the long-term prognosis of these patients and enhance clinical decision-making. The contribution of this study is fourfold. First, we conduct a follow-up study to investigate the occurrence of CKD after three and six months of developing AKI stage 3 in critically ill patients. Second, we employ machine learning techniques to develop predictive models specifically tailored for the CKD prediction task. Third, we extend our analysis to encompass the prediction of mortality in critically ill patients who developed AKI stage 3. Utilizing time-to-event prediction models, we aim to estimate the risk of mortality and provide timely interventions. Moreover, to explore the potential benefits of utilizing unlabeled data, we examine whether the inclusion of additional data from patients who met the inclusion and exclusion criteria but were not included in the follow-up study can improve the accuracy of survival time predictions. By addressing these tasks, our study aims to contribute to the understanding of the long-term outcomes of critically ill patients with AKI stage 3 and provide insights into the development of effective predictive models in the context of CKD and mortality. In the following sections, we will describe the dataset, methodology, experimental results, and provide a comprehensive discussion of our findings. The ultimate goal of this research is to enhance patient care and outcomes through improved risk stratification and personalized interventions based on the prediction of CKD development and mortality in critically ill patients.

## Method

### Study design

Two datasets were used in this study. First, a prospective observational study was conducted using ICU patients aged greater than 18 years who were diagnosed with AKI stage 3 during their stay in AZ Groeninge Hospital in Kortrijk, Belgium, between September 2018 and October 2020. AZ Groeninge Hospital’s institutional review board approved the use of de-identified data in this study (AZGS2018070). All patients have been de-identified and the analyses were conducted in accordance with relevant guidelines and regulations. Participants and/or their legal guardians provided informed consent. Exclusion criteria were patients with a baseline estimated Glomerular Filtration Rate (eGFR) < 30 ml/min/1.73 m$^{2}$ estimated by CKD-EPI^18^, patients with RRT initiated before admission to the ICU, patients with a kidney transplant, patients with therapy restrictions with the shift to palliative care, and patients who received extracorporeal blood purification techniques for reasons other than AKI. During the period of the patient’s stay in the intensive care unit, data were collected using EHR. SCr and cystatin C (CysC) measurements were taken at the time of admission to the ICU and at the time of diagnosis of AKI (in most cases with a short time lag). The patients were also followed up by the nephrology department three, six, nine, and twelve months following diagnosis of AKI stage 3 in the intensive care unit. The eGFR was measured again during these follow-up visits.

In addition to the observational study, we have used an additional dataset containing patients who were not enrolled in this study and as a result, have not been followed up and have no information regarding outcomes. This dataset includes adult patients (> 18 years) who were diagnosed with severe AKI during their ICU stay at AZ Groeninge Hospital in Kortrijk, Belgium, between January 2016 and September 2018 and between October 2020 and September 2021. The exclusion criteria for this dataset were identical to those used in the observational study.

For external validation of our mortality prediction model, we used data from patients who were admitted to the ICU and suffered severe AKI after the observational study ended (between October 2021 and June 2022).

### Acute kidney injury classification

Patients with AKI stage 3, as defined by the KDIGO criteria, have been included in the study. KDIGO defines stage 3 as an increase in SCr up to 3 times from baseline within a 7-day period or urine output (UO) $< 0.3$ *ml*/*kg*/*h* for $\ge 24$ hours^19^. In this study, true baseline SCr was available for patients who had an SCr measurement from an earlier visit (previously to their hospital or ICU admission). When such records were unavailable, baseline SCr was considered the first record of the patient’s hospitalization before ICU admission. All SCr measurements were performed with an Enzymatic method that is traceable to the isotope dilution mass spectrometric method (IDMS), which is the internationally approved reference method for measuring creatinine. In addition, CysC concentrations were measured by Liège University Hospital using a particle-enhanced nephelometric immunoassay on the BNII nephelometer (Siemens Healthcare Diagnostics, Marburg, Germany). The assay was calibrated against the internationally certified reference material ERM-DA471/IFCC for CysC.

### The definition of CKD

We defined CKD as eGFR < 60 ml/min/1.73 m$^{2}$, using the chronic kidney disease epidemiology collaboration (CKD-EPI) corresponding to CKD stage 3 or more according to the KDIGO classification.

### Data description and preprocessing

During the ICU stay, various data points were collected, including demographic information (age, gender, weight, height, and BMI), comorbidity data, details of ICU interventions, the severity of illness scores (APACHE 2), disease classification (SAPS II), sequential organ failure assessment score (SOFA), admission diagnosis, events during the ICU stay (respiratory status, fluid balance, etc.), laboratory data, and kidney function-related features (serum creatinine (SCr), cystatin C (CysC), estimated glomerular filtration rate (eGFR), and albumin measurements), the latter of which was specifically collected for CKD prediction purposes. For certain features like SOFA, SAPS II, and fluid balance, which were measured multiple times during the ICU stay, we calculated the average, maximum value, and change over time (represented as the slope of the fitted line). As patients were transferred to different types of ICUs based on their admission diagnosis (including Medical ICUs (MICUs), Surgical ICUs (SICUs), and Trauma Units), this information was also recorded. The recorded comorbidities encompassed various conditions such as arterial hypertension, chronic liver failure, and diabetes. ICU interventions included the length of invasive ventilation (in days), usage of vasopressors, sedatives, antibiotics, and blood transfusions. In total 52 features (full feature set) have been used for the CKD prediction task. For the mortality prediction task, considering that informed consent was not obtained from the patients for the unlabeled data and their kidney function was not assessed in the same way, this dataset only consists of the features collected from the EHR excluding demographics and kidney-related features, resulting in 30 features (reduced feature set) in total (as the kidney-related features were considered crucial for CKD prediction, we did not consider the unlabeled dataset in that prediction task).

Proper timing of measurements is essential for the accurate prediction of CKD, as different features may have varying levels of relevance at different stages of a patient’s hospitalization. Table 1 lists the features used in our analysis and the corresponding timing of measurement. For example, age, gender, and body weight are measured on the first day of hospital admission, while SOFA and SAPS II are measured on the first day of ICU admission and with one measurement per day. Comorbidities are measured on the first day of hospital admission, while ICU interventions are measured on the first day of ICU admission and with three measurements per day. Our careful consideration of feature timing helps ensure the accuracy and usefulness of our predictive model.

Those features that were missing as a result of incomplete records were imputed by using the Multiple Imputation by Chained Equations (MICE) method^20^ for a maximum of 10 iterations.

**Table 1:** List of features timing.

Feature	Timing
Age	First day of hospital admission
Gender	First day of hospital admission
Body weight	First day of hospital admission
APACHE II	First day of ICU admission
SOFA	First day of ICU admission and one measurement per day
SAPS II	First day of ICU admission and one measurement per day
Comorbidities	First day of hospital admission
ICU interventions	First day of ICU admission and three measurements per day
CKD (after ICU discharge)	Three and six months after AKI
Cystatin C	ICU admission and at the time of developing AKI stage 3
Creatinine	ICU admission and at the time of developing AKI stage 3

### Prediction methods

A random forest (RF)^21^ classifier has been employed to predict CKD after three and six months among patients who developed AKI stage 3 in the intensive care unit. RF is an ensemble learning method that constructs a multitude of decision trees (100 trees in our model) at the time of training and is used for classification, regression, and other tasks. Moreover, feature importance is calculated by weighting the mean decrease in impurity in splits with the given feature by the number of samples in the split^22^.

For our second task (mortality prediction or survival analysis), we assessed two survival methods, random survival forest (RSF)^23^ and survival gradient boosting (XGBoost)^24^. In the XGBoost implementation, there are two different methods for conducting survival analysis, Cox and AFT. In this work, we have used the Cox method. In order to interpret the XGBoost survival model and to show the relative importance of each feature and its effect on the predicting ability, a SHAP (SHapley Additive exPlanations)^25^ summary plot was performed. SHAP is a game theoretic approach that can intuitively and accurately explain the output of a machine learning model. Based on this survival model, the higher the SHAP value, the greater the likelihood of the progression to mortality.

The survival methods were trained on two scenarios of time-to-event data for mortality prediction. The first scenario consists of training the models on the resulting time-to-event data from the observational study (referred to as labeled data, or *Ldata*). This *Ldata* consists of subjects who are either deceased (observed) or alive at the study end or at drop-out (censored). The second scenario consists of adding the unlabeled dataset (referred to as *Udata*) to the first scenario. Due to the absence of information concerning the status of the mortality event (censored or observed) in *Udata*, we set the corresponding status and time equal to zero^26^.

We conducted a 5-fold cross-validation on the CKD prediction datasets in order to estimate the generalization capacity of the models. Results for the mortality prediction task have been evaluated on the external dataset described in “Study design” section .

To better characterize the overall performance of the models, we contrasted the performance of ML algorithms against the conventional statistical methods logistic regression (LR) and Cox proportional hazards model (COXPH)^27^ for CKD and mortality prediction, respectively. We chose logistic regression as a baseline model for classification due to its simplicity, interpretability, and widespread use in the literature. Logistic regression has been extensively applied in medical research and has been shown to perform well in predicting clinical outcomes. Moreover, it has a simple and interpretable model structure, allowing easy clinical interpretation of the results. On the other hand, Cox regression is a commonly used method for survival analysis, which is a type of modeling that accounts for the time until an event of interest occurs. We chose Cox regression over AFT (accelerated failure time) regression in our study because it does not require specifying the baseline hazard function and can handle time-varying variables. In contrast, AFT regression requires specifying the distribution of the survival times, which can be challenging in practice. Additionally, Cox is more widely used in the literature, particularly in medical research.

**Figure 1:**
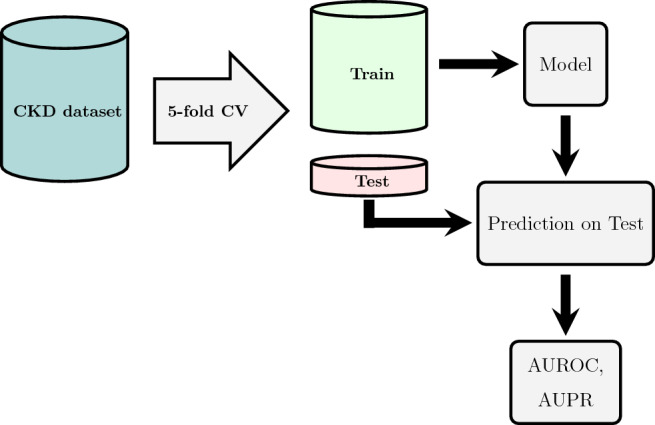
Study workflow for CKD prediction task. We utilized a population of patients from the observational follow-up data to train ML and statistical models to predict CKD after 3 and 6 months of developing AKI stage 3 in the ICU. 5-fold cross-validation was used to train and test models. Prediction performance was assessed with the AUROC and AUPR.

**Figure 2:**
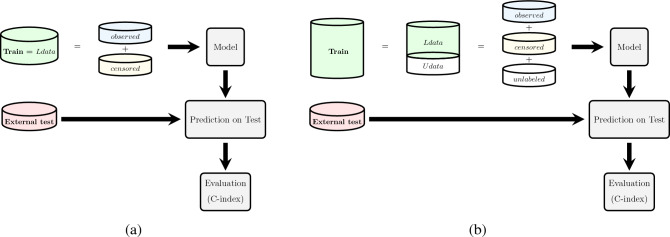
Study workflow for mortality prediction task (survival analysis). (**a**) In the first scenario, we utilized a population of patients from the observational follow-up data (*Ldata*) to train ML and statistical models to predict mortality in patients who developed AKI stage 3 in the ICU. In this scenario, the censoring rate is 57.42%. Prediction performance was assessed using C-index and has been tested on an external test set for each model separately. (**b**) In the second scenario, we utilized a population of patients from the observational follow-up data plus the unlabeled data (*Udata*) to have a bigger training set and train ML and statistical models to predict mortality in patients who developed AKI stage 3 in the ICU. In this scenario, the censoring rate is 80.8%. Prediction performance was assessed using C-index and has been tested on an external test set for each model separately.

The outline of the ML and statistical method workflow is shown for the CKD prediction and survival analysis in Figs. 1 and 2, respectively. Figure 2 represents the two scenarios described earlier.

### Statistical analysis

Categorical features were expressed as numbers (proportions) and continuous features as medians with interquartile ranges (IQR). For the CKD prediction task, the predictive performance of the models was compared using the area under the receiver-operating characteristic curve (AUCROC), the area under the precision-recall curve (AUPR), and the net benefit using the decision curve analysis. AUCROC or ROC curves are constructed by plotting the true positive rate (TPR) against the false positive rate (FPR) at a variety of threshold settings. The true-positive rate is also known as sensitivity or recall, and the false-positive rate is known as (1 - specificity). The AUPR or PR curve illustrates the trade-off between Precision and Recall at various thresholds. In the context of decision curve analysis^28–30^, the net benefit metric serves as a means of comparing the costs and benefits of various treatment approaches. Based on a model’s sensitivity and specificity and the prevalence of the outcome in the population, the net benefit is calculated. According to decision curve analysis, the optimal strategy is the model with the highest utility. Through the cross-validation process, we calculated the predictive performance for each test fold and returned the average over the five test folds. For the mortality prediction task, Harrell’s concordance index (C-index)^31^ which is a common way to evaluate a model in survival analysis, has been used for comparing the predictive performance of the models. All analyses were conducted using Python version 3.9. The XGBoost algorithm was employed for the implementation of the XGBoost model, utilizing the *xgboost* library^32^. The Random Forest (RF) algorithm from the widely-used *scikit-learn* library was utilized^33^. Additionally, the Random Survival Forest (RSF) and Cox proportional hazards (COXPH) models were implemented using the *scikit-survival* library, which provides comprehensive survival analysis capabilities^34^.

### Ethics approval and consent to participate

The study was approved by the institutional review board of AZ Groeninge Hospital (AZGS2018070). Informed consent was obtained by the investigators from the patients or their surrogates before they were enrolled in the study.

## Results

### Patient population

The study included 101 critically ill patients who developed AKI stage 3, with a median age of 74 (IQR 30-92) and 64 (63.3%) males. Characteristics of patients on ICU admission are shown in Table 2. During the study period, 45% of the cohort (n=46) received dialysis for a median of 13 (1-160) days, with a mortality rate of 42.6% (n=43). There were 24 deaths during the ICU stay and 19 deaths during the follow-up phase, including two deaths between the time the patient was discharged from the ICU and the first follow-up. Patient dropouts and mortality resulted in a decrease in the number of patients attending follow-up appointments.

**Table 2:** Population characteristics.

Characteristics	N=101
Demographics	
Age (years), median (IQR)	74 (30 - 92)
Female sex, %	36.6%
Body weight, *kg*	83 (45 - 150)
Body mass index, $kg/m^2$	27.7 (17 - 57)
Scores	
APACHE II	25.9 (11 - 43)
SOFA	9.2 (4.6 - 20.6)
SAPS II	55.2 ( 21.9 - 102.5)
ICU types	
MICU, %	80%
SICU, %	17%
Trauma, %	2%
Comorbidities	
Arterial hypertension, %	48%
Chronic Liver Failure	12%
Diabetes mellitus	22%
Chronic obstructive pulmonary disease	22%
Oncological history	22%
Suspected infection on admission	57%
ICU interventions	
Invasive ventilation days, median (IQR)	0.9 (0 - 20)
Fluid balance, median (IQR)	1032 (182 - 3470)
Transfusion, %	2.2%
Antibiotics, %	88%
Outcomes	
ICU days, median (IQR)	14.5 (1 - 160)
Hospital days, median (IQR)	26 (3 - 186)
CKD (after ICU discharge), %	49%
Mortality (hospital and follow-up), %	43%
Laboratory results	
Cystatin C (mg/L) at ICU admission	1.94 (0.67 - 8.06)
Creatinine (mg/dL) at ICU admission	1.98 (0.31 - 12.64)

For the CKD prediction task, we used data from 101 patients with a full feature set resulting from the observational study. We have removed patients who deceased before three and six months follow-up after AKI, resulting in 75 and 53 patients in the training set, respectively. After three months, 47 of the 75 patients developed CKD, and 33 of the 53 patients developed CKD after six months.

For the mortality prediction task, we used *Ldata* which has 101 subjects and *Udata* with 123 subjects resulting in training the models with 224 subjects in total and with a reduced feature set. Moreover, the external validation set consists of 31 patients with the same reduced feature set.

### Predictive performance: CKD prediction

**Figure 3:**
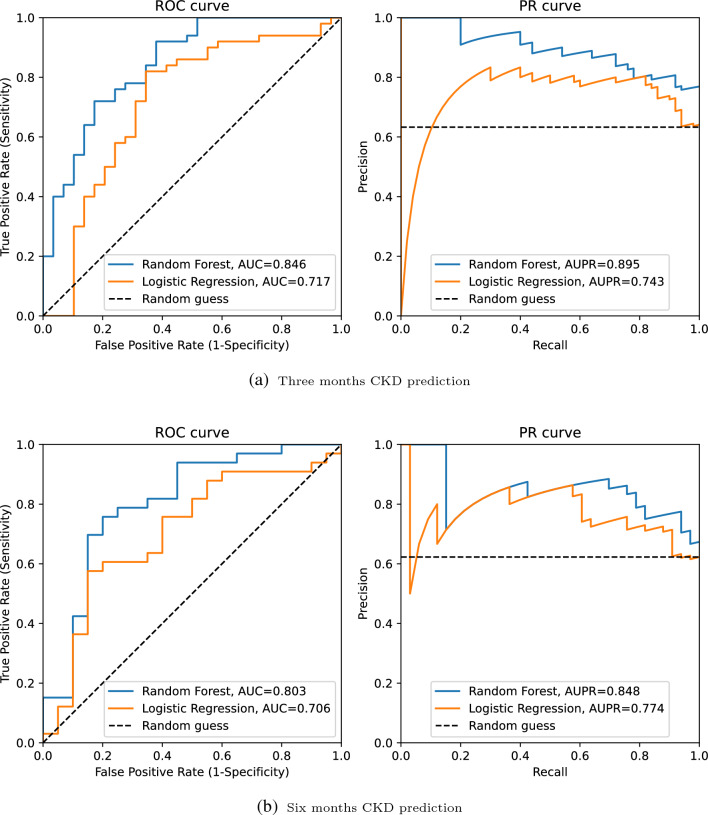
Performance of the Random Forest and Logistic regression model. (**a**) Receiver operating characteristic and Precision-recall curves for estimating the discrimination between the Logistic regression model and the Random Forest model in the prediction of CKD three months after developing AKI. There are 75 subjects in this analysis from whom 63% developed CKD. (**b**) Receiver operating characteristic and Precision-recall curves for estimating the discrimination between the Logistic regression model and the Random Forest model in the prediction of CKD six months after developing AKI. There are 53 subjects in this analysis from whom 62% of them developed CKD.

We performed a comparison between RF and LR for the prediction of CKD after three and six months of developing AKI stage 3, shown in Fig. 3. In both tasks, RF had the highest predictive performance; however, with higher performance after 3 months (AUCROC = 0.846; AUPR = 0.895) compared to predicting CKD 6 months after AKI (AUCROC = 0.803; AUPR = 0.848). There is a reasonable explanation for this performance drop since fewer patients are involved in the second task (53 vs 75) and also, as time passes, patients recover and the ICU-related characteristics may have become less predictive of outcome.

In CKD prediction, we utilized the built-in algorithms for RF models to present feature importance.

**Figure 4:**
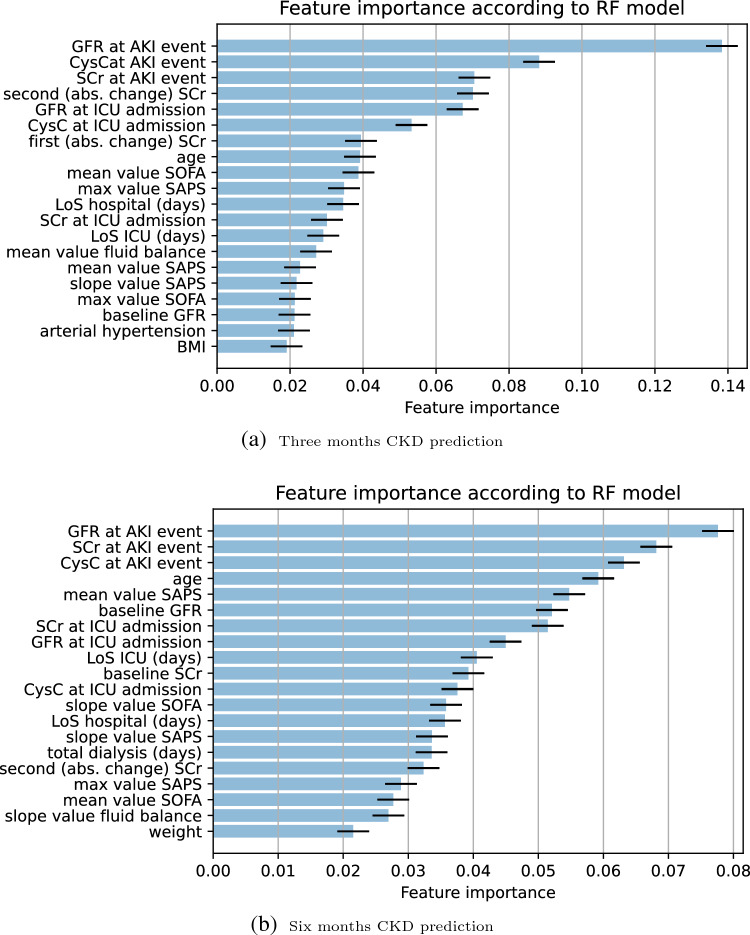
Feature importance for the top 20 features for CKD prediction after prediction. For each classifier, the feature importance estimation was based on the mean decrease in impurity (MDI) calculations. In the features set, first (abs. change) SCr and second (abs. change) SCr show the absolute change in SCr at the ICU admission from baseline SCr and absolute change in SCr at the AKI event from baseline SCr, respectively.

**Figure 5:**
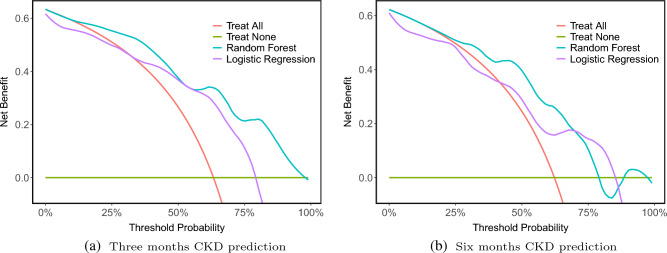
Decision curve analysis graph showing the net benefit against threshold probabilities based on decisions from model outputs. The X-axis indicates the threshold probability for a positive CKD outcome; Y-axis indicates the net benefit.

Figure 4 shows the top 20 features for CKD after AKI predicted by Random Forest models at 3 months and 6 months. According to both models, estimated GFR and creatinine value at the time of the AKI event are the most important features in the prediction of CKD. Besides other kidney-related features (absolute changes in SCr and CysC), the average fluid balance and SOFA score from patients’ ICU stays are also predictive of the development of CKD three months after AKI. In addition, decision-curve analysis shows that compared to the reference model, the net benefit of RF models was larger over all the ranges of clinical threshold, indicating that the RF models prediction would more accurately identify high-risk patients (true-positives) while taking the trade-off with false-positives into consideration. However, the same conclusion cannot be made for LR models as in some thresholds ($\sim$ less than 40%) it is smaller than the clinical threshold (Figure 5).

### Predictive performance: mortality prediction (survival analysis)

We also assessed survival prediction, in two different scenarios (Fig. 2), and made a comparison between three survival models, RSF, XGBoost, and COXPH, and reported the C-index performance on internal (Table 3) and external validation set (Table 4). The standard deviation on the external set was calculated using a bootstrapping technique with 1000 bootstrap samples. The optimal model performance was the survival XGBoost model trained with a combination of labeled and unlabeled data, outperforming all other models with a C-index of 82.48%. Additionally, this XGBoost model demonstrated an increase of 3.48% in predictive performance compared to a model trained only using the labeled data. According to Wilcoxon signed rank test, this increase is significant ($p-value = 0.00087$). Although the RSF model also benefited by 1.42% from adding the unlabeled data to the training set, this increase was the highest for the COXPH model with 11.49%. As a result, our results also confirm that adding unlabeled instances to the training set improves the predictive performance on an independent test set^26^.

**Table 3:** C-index performance on internal validation for mortality prediction.

	COXPH	RSF	XGBoost
Labeled	78.13	91.6	97.19
Labeled + Unlabeled	80.45	95.02	97.24

**Table 4:** C-index performance and the standard deviation on external validation for mortality prediction.

	COXPH	RSF	XGBoost
Labeled	$66.13 \pm 8.48$	$78.9 \pm 7.82$	$79.00 \pm 6.07$
Labeled + Unlabeled	$77.62 \pm 8.05$	$80.32 \pm 8.08$	${82.48} \pm 5.71$

**Figure 6:**
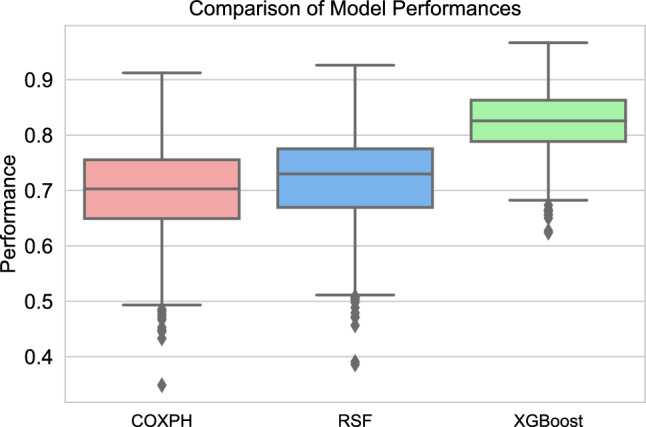
Boxplot comparing model performances of COXPH, RSF, and XGBoost models using both labeled and unlabeled data on the bootstrapped external validation set for mortality prediction.

We also performed a Friedman test to determine if there were significant differences between the predictive performance of the three survival models (COXPH, RSF, and XGBoost) on the bootstrapped external validation set when models used both labeled and unlabeled data for the training. The test revealed a statistically significant difference between the models (Friedman test statistic = 1141.73, $p < 0.001$). To further investigate the differences, we performed pairwise Wilcoxon tests. The results, presented in Table 5, demonstrate statistically significant differences between all pairwise model comparisons. According to the Wilcoxon test, COXPH had a significantly lower predictive performance compared to RSF (Wilcoxon test statistic = 155,434.5, $p < 0.001$) and XGBoost (Wilcoxon test statistic = 8,852.0, $p < 0.001$). Additionally, RSF exhibited a significantly higher predictive performance compared to COXPH (Wilcoxon test statistic = 12,561.0, $p < 0.001$). Moreover, XGBoost showed a significantly higher predictive performance compared to both COXPH (Wilcoxon test statistic = 155,434.5, $p < 0.001$) and RSF (Wilcoxon test statistic = 12,561.0, $p < 0.001$). Based on these findings, we conclude that the XGBoost and RSF models outperformed the COXPH model in predicting the target variable. The boxplot (Fig. 6) visually represents the distribution of performance values for each model, indicating that XGBoost had the highest median performance among the three models.

**Table 5:** Pairwise Wilcoxon test results for model comparisons.

Model comparison	Wilcoxon test statistic	*P* value
COXPH vs RSF	155434.5	1.009718e-20
COXPH vs XGBoost	8852.0	7.260816e-154
RSF vs XGBoost	12561.0	7.039896e-149

**Figure 7:**
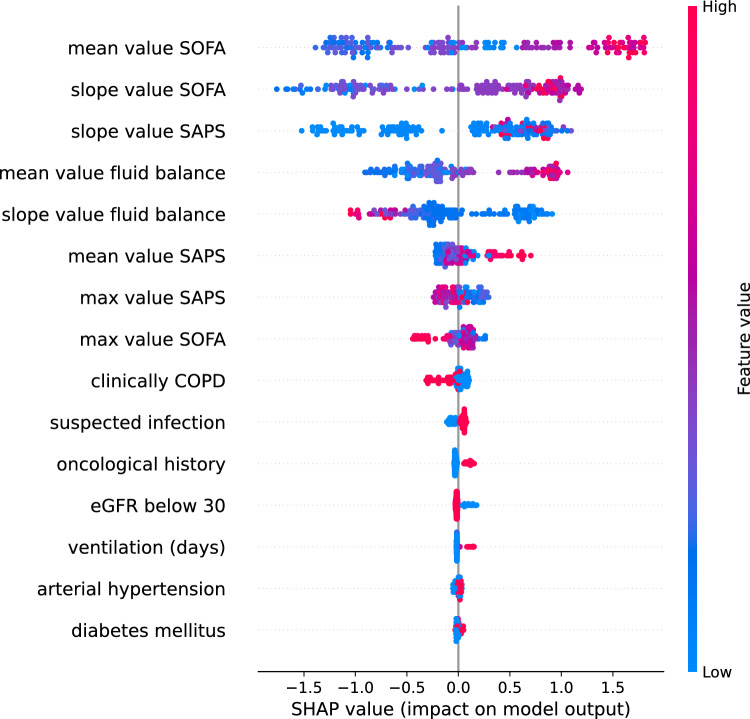
SHAP value of XGBoost model output. Each point represents a variable together with an observation. As demonstrated by the color bar, higher values are shown in red, while lower values are shown in blue.

Figure 7 shows the SHAP values of XGBoost (the best-performing survival model). For each feature, one point represents one patient. Positions along the x-axis represent the impact a feature has had on the model’s output for that specific patient. Mathematically, this corresponds to the (logarithm of the) mortality risk relative across patients (i.e., a patient with a higher SHAP value has a higher mortality risk relative to a patient with a lower SHAP value). On the y-axis, features are arranged in order of importance, which is determined by the mean of their absolute Shapley values. A feature’s position in the plot indicates its importance for the model. According to the SHAP, the average value and the evolution of severity of illness scores (SOFA and SAPS) during the ICU stay are important mortality risk factors for patients who experienced severe AKI. Among other features, the average amount of fluid balance contributes significantly to the prediction of mortality in AKI patients. However, the slope value for fluid balance seems to contribute adversely. A possible explanation could be that during the acute phase of critical illness, the fluid balance tends to be more positive after a few days (compared with the days before). Fluid balance “positivity” decreases as a patient’s condition improves, eventually becoming negative. An intensivist usually begins diuretics or initiates dialysis when (s)he observes a daily increase in fluid balance positivity. It may lead to a more rapid return to normal fluid balance, or even a negative fluid balance (which is what diuretics are intended to accomplish).

## Discussion

### Machine learning for improved clinical decision-making

Due to the extensive use of EHRs as well as recent advances in machine learning, AI is expanding its influence on healthcare and has gradually changed the manner in which clinicians approach problem-solving^35^. However, to our best knowledge, there has not been any attempt to apply ML methods to predict the occurrence of CKD in AKI patients. In this study, we evaluated the potential utility of machine learning as a tool to improve clinical decision-making in critically ill patients who experienced AKI stage 3. Specifically, we addressed two important prediction tasks: the development of CKD and mortality.

### Prediction of CKD after severe AKI

First, prediction models for CKD in critically ill patients after three and six months of experiencing severe AKI were explored using the random forest algorithm. In addition, as a time-to-event analysis, we have predicted mortality in the same group of patients using two different survival models, namely random survival forest and survival XGBoost. In the CKD prediction task, the RF models had excellent performance with an AUPR of 0.895 and 0.848 for three months and six months CKD, respectively, which was significantly better than the performance of the baseline logistic regression model (0.743 and 0.774, respectively). The results of variable importance ranking can also potentially inform clinical practice. In our analysis, the importance of creatinine and cystatin C at the time of developing AKI was determined by interpreting the importance of each variable in the RF models to predict CKD. In addition, the decision curve analysis indicates that these models show a benefit compared to the ’treat everyone’ and ’treat none’ approaches, which indicates the possibility of allocating targeted assessments and interventions in addition to those broad health service strategies. Overall, our results in predicting CKD after severe AKI showed that machine learning models are tools that can be helpful in clinical decision-making. The presented ML models can be quite useful in practice since they are based on features that are routinely collected in ICU and laboratory data, which are easily assessable in most ICU units. In addition, the need for early detection and prevention of CKD is important. However, currently, after discharge from ICU, the follow-up of AKI survivors is considerably challenging mainly due to being time-consuming and costly, and patients drop out. As a result, using the presented ML models, a risk profile can be developed for each survivor of AKI using electronic health records (EHR) data upon discharge from the ICU, which allows clinicians to create a customized follow-up plan.

### Prediction of mortality in AKI patients

We extended our analysis to address the prediction of mortality in critically ill patients who developed AKI stage 3. We utilized two different survival models, namely random survival forest (RSF) and survival XGBoost. The survival XGBoost model had a significantly higher performance with a c-index of 0.79 in predicting mortality compared to the baseline COXPH model with a c-index of 0.661. Moreover, the performance of the XGBoost model further improved significantly when incorporating a set of baseline data (unlabeled data) with no information about mortality, reaching a c-index of 0.824. The Friedman test revealed that there were statistically significant differences in predictive performance among three survival models (COXPH, RSF, and XGBoost) on an external validation set when using labeled and unlabeled data for training. Post-hoc tests confirmed that XGBoost and RSF outperformed Cox in predicting mortality ($p < 0.001$).

### Utilizing unlabeled data for improved survival predictions

Follow-up studies in clinical settings often experience a reduction in data collection, which can complicate subsequent analyses. On the other hand, there is often a substantial amount of baseline data available on patients with similar characteristics and background information, for example, from patients outside the study time window. Our analysis has shown that such unlabeled data instances can be used to predict survival times with a high degree of accuracy. Adding unlabeled data with censoring time and status equal to zero may appear counterintuitive in the context of the Cox model, as the learned parameters are not affected by such observations. However, it is important to note that the baseline hazard function in the Cox model can be influenced by the inclusion of additional data points, even with zero event times. The baseline hazard represents the underlying risk of an event occurring in the absence of any predictors, and incorporating unlabeled data can refine its estimation. Although the learned parameters remain unchanged, the additional unlabeled data may provide valuable insights into the shape and variability of the baseline hazard function. These insights can enhance the model’s ability to capture the underlying risk patterns, which could lead to improved predictive performance. While further investigation and discussion are required to fully interpret these findings, it is plausible that the unlabeled data contribute to a more accurate estimation of the baseline hazard, thus enhancing the Cox model’s predictive capabilities. Additionally, adding unlabeled data with censoring time and status equal to zero does not have a significant impact on the performance of Random Survival Forest (RSF) models. The reason for this is that the resulting ensembles are very similar, and the trees generated by RSF are contained in the trees generated by RSF with unlabeled data. This is because the addition of censored data points with event time set to zero does not influence the log-rank splitting criterion, which is used by RSF to build trees. However, the size of the trees may be affected by the addition of censored data points. The impact of unlabeled data on the performance of survival XGBoost can vary depending on the dataset and the specific implementation of XGBoost. In general, adding unlabeled data can potentially improve the performance of XGBoost models by providing additional information for the algorithm to learn from. However, if the unlabeled data is biased or not representative of the true distribution of the survival times, it could also harm the performance of the model. It is important to carefully evaluate the impact of unlabeled data on the performance of the model and consider the potential risks and benefits before incorporating it into the analysis. In our analysis, we found that adding unlabeled data improved the performance of all three models, Cox, RSF, and survival XGBoost, not only in the validation set but also in an external test set. This indicates that the addition of unlabeled data did not introduce noise or bias into the models and suggests that our choice of the unlabeled set was appropriate. The improvement in performance across multiple models provides further support for the usefulness of incorporating unlabeled data in survival analysis to improve predictive accuracy. In light of these findings, ML seems to be a viable approach to predicting CKD progression and mortality in AKI patients, which may help physicians establish personalized treatment plans at an early stage.

### Implications and limitations

The study we conducted has a number of strengths. First, we examined not only discrimination but also clinical usefulness, which was considered a key measure of the performance of the model. In addition, we also validated the prediction model for our primary outcome of mortality in a distinct cohort of critically ill patients who developed AKI. Furthermore, the explanations and interpretations that accompany the predicted chance of CKD and the overall survival are intended to give clinicians insights regarding how each prognostic factor contributes to CKD development and overall survival. However, several limitations should be considered. First, the small sample size is a limitation of this study because it might have affected the generalizability and statistical power of the models. A larger dataset would have allowed for more robust analysis and might have revealed more subtle relationships between variables. Moreover, the small sample size might have led to overfitting, which can cause the models to perform well on the training data but poorly on new, unseen data. Therefore, the findings of this study should be interpreted with caution and validated on larger, independent datasets before being used in clinical practice. A second limitation is the fact that the data for our models were provided by a single center, which may have compromised their generalizability. Additionally, the use of unlabeled data may violate the assumption of non-informative censoring, and the unlabeled set should be carefully chosen to avoid incorporating a biased set that could introduce noise into the algorithm. Therefore, further research is needed to investigate the potential impact of adding unlabeled data on the results and to identify appropriate methods for selecting unlabeled data to ensure its usefulness and avoid introducing bias. This research could also explore the impact of adding different types of unlabeled data, such as data with censored status and event times greater than zero, on the performance of survival models. By further investigating the use of unlabeled data in survival analysis, we can better understand its potential benefits and limitations and develop best practices for incorporating this type of data into predictive models.

## Conclusion

According to our study, machine learning models are able to improve the prediction of CKD and mortality in critically ill patients who developed severe AKI during their stay in the intensive care unit. Our presented models had excellent performance in predicting CKD and mortality and were significantly better than the performance of the baseline models. In addition, we have investigated the inclusion of unlabeled data points in the survival analysis task. More precisely, we have shown that learning from data with three degrees of supervision: fully observed, partially observed (censored), and unobserved (unlabeled) data points lead to better performance in mortality prediction. Although our time-to-event models have been tested on an external validation set, the CKD prediction models need to be tested externally.

## Data Availability

The datasets used and/or analyzed during the current study are available from the corresponding author upon reasonable request.

## Abbreviations

AKI: Acute kidney injury
CKD: Chronic kidney disease
ICU: Intensive care unit
ML: Machine learning
SCr: Serum creatinine
CysC: Cystatin C
AKD: Acute kidney disease
RRT: Renal replacement therapy
EHR: Electronic health record
IDMS: Isotope dilution mass spectrometric method
CKD-EPI: Chronic kidney disease epidemiology collaboration
eGFR: Estimated Glomerular Filtration Rate
UO: Urine output
MICUs: Medical ICUs
SICUs: Surgical ICUs
RF: Random forest
RSF: Random survival forest
SHAP: Shapley additive explanations
MICE: Multiple imputation by chained equations
LR: Logistic regression
COXPH: Cox proportional hazards
IQR: Interquartile ranges
AUCROC: Area under the receiver-operating characteristic curve
AUPR: Area under the precision-recall characteristic curve
TPR: True positive rate
FPR: False positive rate
C-index: Concordance index
MDI: Mean decrease in impurity
HSD: Honestly significant difference
FWER: Family-wise error rate.

## Code availability

The code used to train and validate the model will be made available at https://github.com/FatemeNateghi.
